# The ancient E-ring in bacterial flagellar motors

**DOI:** 10.1093/femsre/fuag011

**Published:** 2026-03-12

**Authors:** Siqi Zhu, Xueyin Feng, Yanran Liu, Wei Hu, Beile Gao

**Affiliations:** State Key Laboratory of Tropical Oceanography, Guangdong Provincial Key Laboratory of Applied Marine Biology, Guangdong Provincial Observation and Research Station for Coastal Upwelling Ecosystem, South China Sea Institute of Oceanology, Chinese Academy of Sciences, Guangzhou 510301, China; Sanya National Marine Ecosystem Research Station, Tropical Marine Biological Research Station in Hainan, Chinese Academy of Sciences, and Key Laboratory of Tropical Marine Biotechnology of Hainan Province, Sanya 572000, China; State Key Laboratory of Tropical Oceanography, Guangdong Provincial Key Laboratory of Applied Marine Biology, Guangdong Provincial Observation and Research Station for Coastal Upwelling Ecosystem, South China Sea Institute of Oceanology, Chinese Academy of Sciences, Guangzhou 510301, China; Sanya National Marine Ecosystem Research Station, Tropical Marine Biological Research Station in Hainan, Chinese Academy of Sciences, and Key Laboratory of Tropical Marine Biotechnology of Hainan Province, Sanya 572000, China; University of Chinese Academy of Sciences, Beijing 100049, China; State Key Laboratory of Tropical Oceanography, Guangdong Provincial Key Laboratory of Applied Marine Biology, Guangdong Provincial Observation and Research Station for Coastal Upwelling Ecosystem, South China Sea Institute of Oceanology, Chinese Academy of Sciences, Guangzhou 510301, China; Sanya National Marine Ecosystem Research Station, Tropical Marine Biological Research Station in Hainan, Chinese Academy of Sciences, and Key Laboratory of Tropical Marine Biotechnology of Hainan Province, Sanya 572000, China; University of Chinese Academy of Sciences, Beijing 100049, China; State Key Laboratory of Microbial Technology, Shandong University, Qingdao 266237, China; State Key Laboratory of Tropical Oceanography, Guangdong Provincial Key Laboratory of Applied Marine Biology, Guangdong Provincial Observation and Research Station for Coastal Upwelling Ecosystem, South China Sea Institute of Oceanology, Chinese Academy of Sciences, Guangzhou 510301, China; Sanya National Marine Ecosystem Research Station, Tropical Marine Biological Research Station in Hainan, Chinese Academy of Sciences, and Key Laboratory of Tropical Marine Biotechnology of Hainan Province, Sanya 572000, China

**Keywords:** flagella, motility, E-ring, scaffold, motor, evolution

## Abstract

The bacterial flagellum is an elaborate nanomachine that powers motility in a variety of environments. While recent cryo-electron tomography studies have revealed great complexity as well as diversity in flagellar motor structures, less is known about the components that constitute the auxiliary structures observed in the periplasm for several species. One example is the E-ring, which was first observed in 1979 in *Caulobacter crescentus* but whose composition has only recently been shown to be a single protein, FlgY and its homologs. Multiple FlgY dimers form a conserved ring-spoke structure encircling the MS-ring, although the impact of the E-ring on motility seems to differ across bacterial phyla. Remarkably, the E-ring is widely present in flagellated species in the *Bacteria* domain except β- and γ-proteobacteria, suggesting an ancient origin that likely traces back to the last bacterial common ancestor. Future investigation is required to determine the exact role of this conserved structure in motor function, which may reveal mechanisms distinct from the current working model based on *Escherichia coli* and *Salmonella enterica*, which lack the E-ring, and also shed light on the architecture and function of the ancestral motor.

## Teaser

E-ring is a “ring and spoke” structure encircling the upper part of the MS-ring in bacterial flagellar motors. This structure, made of FlgY homologs, is ancient and prevalent in flagellated bacteria but functions distinctively among different species.

## Introduction

The flagellum is a complex nanomachine used by bacterial cells to drive motility and also the first identified rotary motor in biology (Berg and Anderson 1973). It has long attracted scientists from diverse disciplines to study its assembly, rotational mechanisms, and evolution (Armitage and Berry 2020, Beeby et al. 2020). Bacterial flagellum is a cell surface appendage, with a basal body embedded in cell envelope as well as extracellular hook, hook-filament junction, filament, and filament cap (Johnson et al. 2021, Tan et al. 2021, Einenkel et al. 2025, Guo et al. 2025). To make such a trans-envelope structure, more than 50 genes in bacterial genomes are involved in its regulation and self-assembly (Chevance and Hughes 2008). How the flagellar motor rotates and how a bidirectional motor switches between clockwise and counterclockwise directions remain as fundamental questions of significant interest (Chang et al. 2020, Hu et al. 2022, Johnson et al. 2024, Singh et al. 2024, Tan et al. 2024).

**Figure 1:**
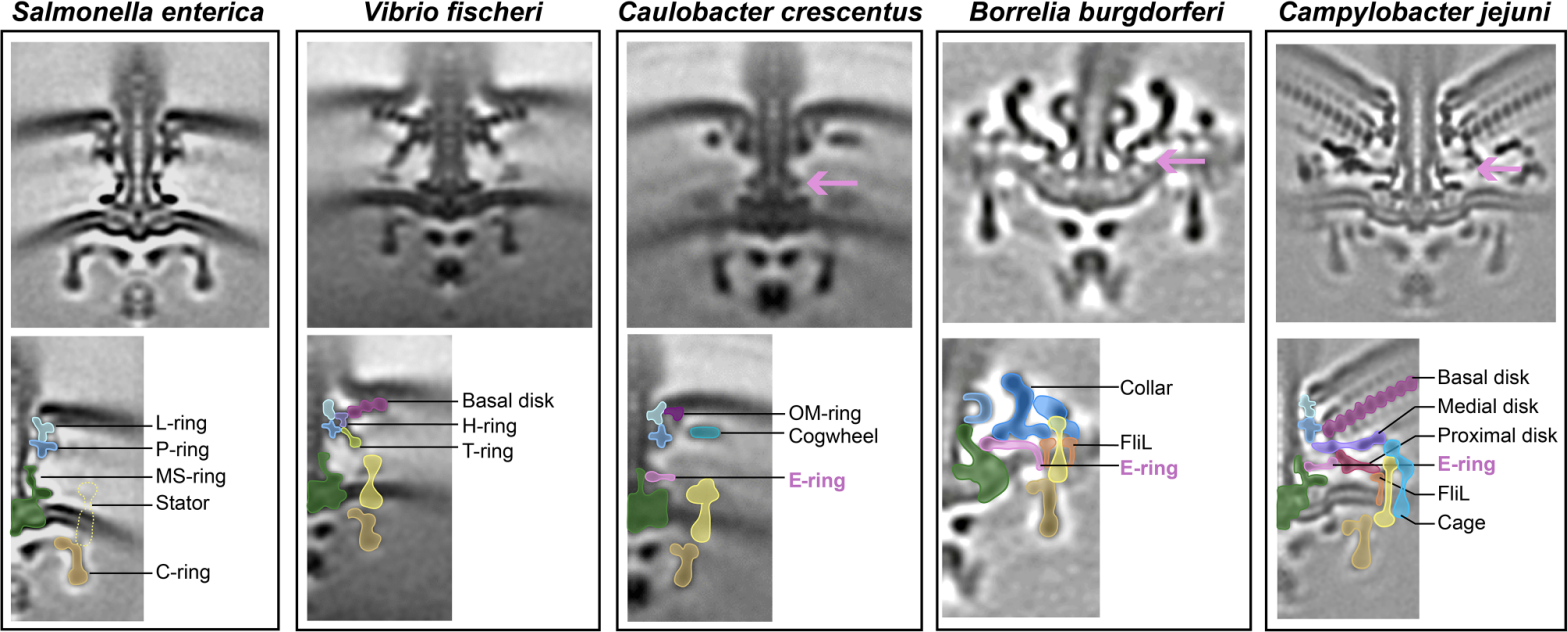
*In situ* structures of flagellar basal bodies/motors from different species. Upper panels show intact flagellar motors and the potential E-ring is indicated by arrow. Lower panels show cartoon representations highlighting conserved ring structures and species-specific periplasmic scaffolds. The dotted line in the lower panel of *S. enterica* motor indicates that the stator units are not visible due to dynamic exchange, in contrast to the visible and fixed number of stator units in the other four species on its right. Cryo-ET images taken from: *S. enterica* (Zhu et al. 2019); *Vibrio fischeri* (Beeby et al. 2016); *Caulobacter crescentus* (Rossmann et al. 2020); *Borrelia burgdorferi* (Liu et al. 2009); and *Campylobacter jejuni* (Feng et al. 2026).

*Escherichia coli* and *Salmonella enterica* have been used as model organisms in flagellar studies for decades (Wadhwa and Berg 2022). Pioneer studies of flagellar structure examined purified flagella by electron microscopy (EM) since 1960s, which provide the foundation to understand its functional mechanism (Abram et al. 1965). At a resolution that is not comparable to today’s single-particle cryo-EM, the flagellar basal bodies could be discerned to consist of a rod and several rings (DePamphilis and Adler 1971). In the classical *E. coli* model, four rings surround the rod: the L-ring (**l**ipopolysaccharide ring), P-ring (**p**eptidoglycan ring), MS-ring (**m**embrane-**s**upramembrane ring), and C-ring (**c**ytoplasmic ring) (Fig. 1). The L-ring and P-ring serve as bushings that support low-friction rotation of the rod, whereas the MS-ring and C-ring form the rotor at the base of the rod (Johnson et al. 2020, Kawamoto et al. 2021, Yamaguchi et al. 2021). The cytoplasmic part of the rotor interacts with transmembrane stator units, which are protein complexes made of MotA_5_B_2_. Recent single-particle cryo-EM studies suggest that the stator units can harness the ion motive force to rotate themselves, then generate torque to rotate the rotor as well as the rod and the extracellular hook and filament (Deme et al. 2020, Santiveri et al. 2020).

The development of cryo-electron tomography (cryo-ET) opened a new window for visualization of the flagellar basal bodies *in situ*, with the first motor structure within intact cells published in 2006 (Murphy et al. 2006). A whole new world of motors with great structural diversity and unprecedented complexity compared to the *E. coli* paradigm in a range of bacterial species has since been revealed (Chen et al. 2011, Zhao et al. 2013, Zhu et al. 2019). Specifically, in addition to the highly conserved structure of the classic *E. coli* model, the basal body of many species includes a variety of periplasmic decorations surrounding the central rod. For example, non-enteric γ-proteobacteria such as species from genera *Vibrio, Pseudomonas, Shewanella*, and *Aeromonas* possess both H-ring and T-ring (Molero et al. 2011, Merino and Tomas 2016, Zhu et al. 2017, Zhu et al. 2018, Zhu et al. 2019) (Fig. 1); and one α-proteobacterial species *Cereibacter sphaeroides* was also shown to encode homologs for H-ring components (Fabela et al. 2013, Perez-Gonzalez et al. 2019). In addition, α-proteobacterial *Caulobacter crescentus* has a cogwheel-like structure (Rossmann et al. 2020); *Spirochaetota* species with periplasmic flagella (endoflagella) have a unique collar structure (Xu et al. 2020, Chang et al. 2021); *Campylobacter jejuni* and *Helicobacter pylori* that belong to the *Campylobacterota* phylum (previously called ε-proteobacteria) have several large periplasmic disks and a peripheral cage (Beeby et al. 2016, Liu et al. 2024) (Fig. 1). In contrast, the *E. coli* motor appears to be the simplest among those examined by cryo-ET to date (Fig. 1), thus it has been proposed as the prototype of ancestral motor due to its structural simplicity (Chaban et al. 2018, Rossmann and Beeby 2018).

The auxiliary periplasmic scaffolds in complex motors are mostly related to stator stability and believed to be products of species-specific adaptations to diverse environmental conditions (Cohen et al. 2024, Liu et al. 2024, Drobnic et al. 2025). For example, the H-ring and T-ring in *Vibrio* spp. play important role in anchoring the sheathed flagellum to the outer membrane and also in recruiting sodium-driven stator units to enable fast swimming (Zhu et al. 2017, 2018). In *C. jejuni* motor, the basal disk made of FlgP is essential for the assembly of stator ring and other periplasmic disks thus required for motility (Beeby et al. 2016). Further studies suggest that the basal disk acts as a flange to brace the flagellar motor during disentanglement of flagellar filament from interactions with the cell body and other filaments, consistent with the fact that the cell surface and flagellar filament of *C. jejuni* are heavily glycosylated (Szymanski and Gaynor 2012, Cohen et al. 2024). However, the function or mechanism of many auxiliary scaffolds in flagellar motors of various species remain unknown, which are avenues for future research.

**Figure 2:**
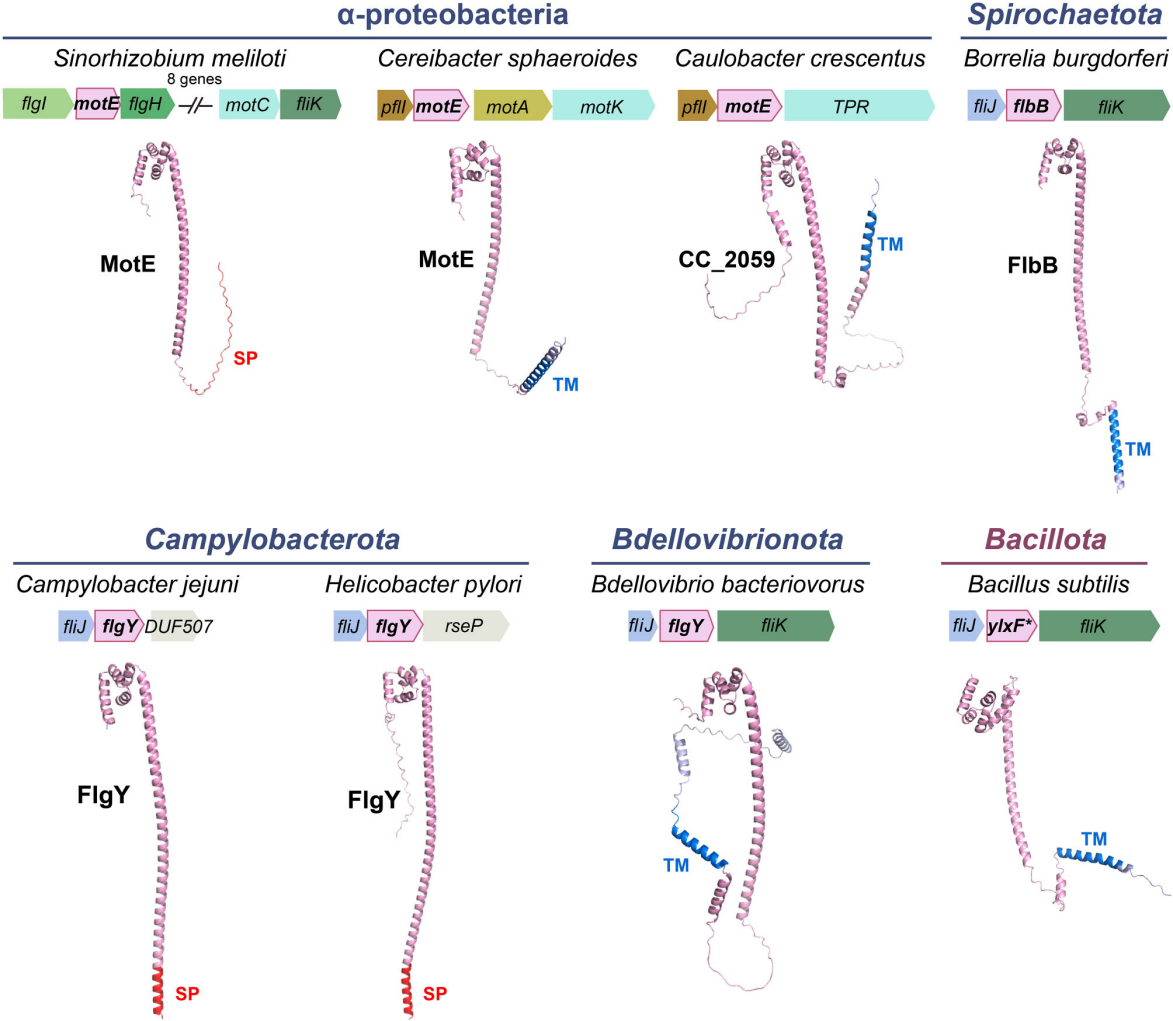
Structures and genomic contexts of candidate components of E-ring across species. All structures are predicted by AlphaFold 3 (Abramson et al. 2024). The transmembrane motif (labled as TM) and signal peptide (labeled as SP) are predicted by TMHMM 2.0 (Krogh et al. 2001) and SignalP 6.0 (Teufel et al. 2022) , respectively. Gene *ylxF* in *Bacillus subtilis* is labeled by * in order to highlight its commonly used gene name in the model organism of monoderm bacteria for flagellar studies. In addition, *Bacillota* (previously *Firmicutes*) is distinguished as the only phylum from Terrabacteria, whereas all the other phyla belong to Gracilicutes.

Here, we summarize studies on the E-ring of the flagellar motor, which was discovered four decades ago but its constituent protein has only been identified very recently (Johnson et al. 1979, Feng et al. 2026). Due to great sequence divergence and phenotypical differences related to mutant strains, the sole component of E-ring has been named differently across bacterial phyla: e.g. MotE in α-proteobacteria, FlbB in *Spirochaetota*, and FlgY in *Campylobacterota* (Fig. 2) (Eggenhofer et al. 2004, Moon et al. 2016, Velez-Gonzalez et al. 2024, Feng et al. 2026). The evolutionary and functional relatedness among MotE/FlgY/FlbB was recently established based on their conserved structural fold and their inclusion in the MotE orthologous group (COG3334) (https://www.ncbi.nlm.nih.gov/research/cog/cog/COG3334/) (Botting et al. 2025, Tachiyama et al. 2025, Feng et al. 2026). In this regard, the distinctive “ring-spoke” structure that FlgY and FlbB build around the MS-ring in the motor suggests that a similar ring may also be present in species harboring a MotE homolog. In this review, we provide an overview of the biochemical and physiological aspects of these proteins. Furthermore, we highlight the structural variations of the E-ring across bacterial phyla, revealing its ancient origin and widespread presence in the *Bacteria* domain.

## The discovery of the E-ring in *C. crescentus*

The E-ring was first discovered in purified flagellar basal bodies from *C. crescentus* by EM in 1979 (Johnson et al. 1979). A small, thin disk was found between P-ring and MS-ring, and was named the “**E**xtra **ring**” since it was not observed in motor structure of the model organism *E. coli* (Stallmeyer et al. 1989). The E-ring was hypothesized to be involved in flagellar ejection, which is a unique feature of *C. crescentus* during its life cycle, although there was no experimental evidence supporting this hypothesis (Johnson et al. 1979). Later, EM observation of purified basal bodies from a *C. crescentus* Δ*flaD* mutant (later reannotated as Δ*flgA* to align with the *E. coli* nomenclature and FlgA is a chaperone for the P-ring assembly), revealed only partial motor assembly (Hahnenberger and Shapiro 1987, Hahnenberger and Shapiro 1988). Notably, this partial motor structure from the Δ*flgA* mutant consisted of a rod, the MS-ring, and the E-ring, suggesting that the E-ring is not only assembled early but is also a stable structure that can be purified together with the central rod.

Recently, *in situ* cryo-ET studies of the *C. crescentus* motor confirmed the presence of this additional ring just above the MS-ring (Fig. 1C) (Rossmann et al. 2020). A similar ring structure at the same location was also observed in motor structures of four species from other taxa: *C. jejuni, H. pylori*, and *Wolinella succinogenes* of the phylum *Campylobacterota* and *Bdellovibrio bacteriovorus* of the phylum *Bdellovibrionota* (previously a lineage of δ-proteobacteria) (Rossmann et al. 2020). Although the E-ring seemed to be widely present based on cryo-ET observation, its composition was unknown in all these species.

## MotE in *Sinorhizobium meliloti* and *C. sphaeroides*

The α-proteobacteria *Sinorhizobium meliloti* and *C. sphaeroides* both possess a flagellar protein belonging to the COG3334 group that has been genetically and biochemically characterized. In the soil bacterium *S. meliloti*, additional flagellar components MotE and MotC were identified to be required for its motor function (Table 1) (Eggenhofer et al. 2004). Deletion of *motE* resulted in degradation of the periplasmic protein MotC, and MotE binds to MotC (Eggenhofer et al. 2004). In addition, MotE bears an N-terminal signal peptide (Fig. 2) and is rapidly secreted into the periplasm, where it forms stable dimers (Eggenhofer et al. 2004). Dimerization is essential for its stability in the periplasm and protein function. Thus, MotE was suggested to act as a chaperone for MotC folding (Eggenhofer et al. 2004).

**Table 1:** Comparison of E-ring homologs across diverse species.

Taxa	Organisms	Homologs (length/aa)	Knockout penotypes	Interaction proteins	E-ring symmetries	Flagellation patterns	References
α-proteobacteria	*S. meliloti*	MotE (179)	Non-motile, flagellated, MotC levels significantly reduced.	MotC	Unknown	Peritrichous	(Eggenhofer et al. 2004)
	*C. sphaeroides*	MotE (185)	Non-motile, flagellated.	MotK, MotB	Unknown	Polar and subpolar	(Vélez-González *et al*. 2024)
	*C. crescentus*	MotE (264)	Unknown.	Unknown	Unknown	Polar	None
*Spirochaetota*	*B. burgdorferi*	FlbB (205)	Non-motile, flagellated, abnormal periplasmic flagella orientation, rod-shape, FlaB levels reduced, lack of collar/stator/FliL.	FliL, FlcA, FlcB, FlcC, FlcD, FliF	16	Polar	(Moon et al. 2016, Moon et al. 2018, Xu et al. 2020, Chang et al. 2021, Botting et al. 2025)
*Campylobacterota*	*C. jejuni*	FlgY (172)	Motile, flagellated, invasion defect, lack of E-ring.	PflA	17	Polar	(Gao et al. 2014, Feng et al. 2026)
	*H. pylori*	FlgY (219)	Less motile, flagellated, lack of E-ring/distal spokes, unstable cage/distal ring/stator.	PflA, PflB	13	Polar	(Tachiyama et al. 2025)

A homolog of MotE was recently studied in *C. sphaeroides*, which possess two distinct flagellar systems (Table 1) (Velez-Gonzalez et al. 2024). The *fla1* genes encode components for the assembly of a single subpolar flagellum, whereas the *fla2* gene products assemble several polar flagella (Hernandez-Valle et al. 2017). MotE is essential for the polar flagellar rotation in *C. sphaeroides*, and it interacts with MotK, rather than MotC, which is believed to be missing in this species (Velez-Gonzalez et al. 2024). Both *motE* and *motK* are located in the *fla2* gene cluster only, not found in the *fla1* gene cluster, and their gene products interact with MotB2 (Velez-Gonzalez et al. 2024). Thus, it was proposed that the paralyzed flagellum phenotype of Δ*motE* and Δ*motK* mutants might be due to defect in recruitment or functioning of stator units (Velez-Gonzalez et al. 2024). Interestingly, both MotC of *S. meliloti* and MotK of *C. sphaeroides*, are mainly composed of **t**etratrico**p**eptide **r**epeats (TPR), except that MotK has an additional peptidoglycan-binding AMIN (**ami**dase **N**-terminal) domain at its N terminus (Velez-Gonzalez et al. 2024). Hence, it is possible that both MotC and MotK interact with MotE by their shared TPR repeat.

Despite the above findings, no direct evidence is yet available showing whether MotE is a chaperone or a structural component of the motor, since no cryo-ET studies have been performed on the motors of these two species. Although MotE stabilizes MotC in *S. meliloti*, this does not exclude the possibility that MotE could be part of motor structure (Eggenhofer et al. 2004). A similar situation has been reported for the MotAB stator complex, for which the stable existence of MotB is dependent on the presence of MotA (Wilson and Macnab 1990). Moreover, sfGFP fused MotE co-localizes with the flagellar structure in *C. sphaeroides*, and that this localization does not require MotK, MotA2, or MotB2, arguing against a chaperone-only role (Velez-Gonzalez et al. 2024). Collectively, these observations reinforce the possibility that MotE is a structural component of the E-ring.

## FlbB in *Borrelia burgdorferi*

The endoflagella of *Borrelia burgdorferi* have been extensively studied due to their exceptionally complex structure within the confined periplasmic space and the important role of flagellar motility in spirochaete pathogenesis (Motaleb et al. 2015, Sultan et al. 2015, Wunder et al. 2016, Zamba-Campero et al. 2024). Only species of the *Spirochaetota* phylum are known to have endoflagella and all examples examined by cryo-ET to date possess a unique collar around the central rod, albeit with a distinct shape in different genera of this phylum (San Martin et al. 2023). The collar structure is large, anchored to the inner membrane and the MS-ring, and interacts with the stator units (Fig. 1) (Xu et al. 2020, Chang et al. 2021). In the past decade, studies led by Dr. Jun Liu in several laboratories have identified several building blocks of the collar using *B. burgdorferi* as a model. FlbB was the first identified collar protein, followed by four other components FlcA, FlcB, FlcC, and FlcD (BB0236) (Moon et al. 2016, 2018, Xu et al. 2020, Chang et al. 2021, Xu et al. 2021). According to the most recent model, there are still other yet unknown proteins needed to fulfill the known structure of the large collar complex (Botting et al. 2025).

The *flbB* gene is encoded within a flagellar operon and was thought to be spirochete-specific, since its protein sequence shares no significant similarity with proteins from outside of this phylum (Moon et al. 2016). Deletion of *flbB* led to loss of motility and a change in morphology from the iconic flat-wave shape to a rod shape (Table 1) (Moon et al. 2016). In addition, the Δ*flbB* mutant displayed an abnormal periplasmic flagellar orientation toward the cell pole, in contrast to the inward orientation toward the cell center in the wild type (Moon et al. 2016). Importantly, cryo-ET and subtomogram averaging analyses revealed that the collar structure is totally absent in a Δ*flbB* mutant, and the 16 stator units, as well as the FliL structures, also disappear (Moon et al. 2016). Furthermore, examination of GFP fused FlbB by cryo-ET suggested that FlbB is located at the base of the collar and is anchored to the inner membrane, which is consistent with the existence of a transmembrane motif at the N-terminus of FlbB (Fig. 2) (Moon et al. 2016). Altogether, these data suggest that FlbB is essential for collar assembly, stator loading, proper orientation and function of endoflagellum.

**Figure 3:**
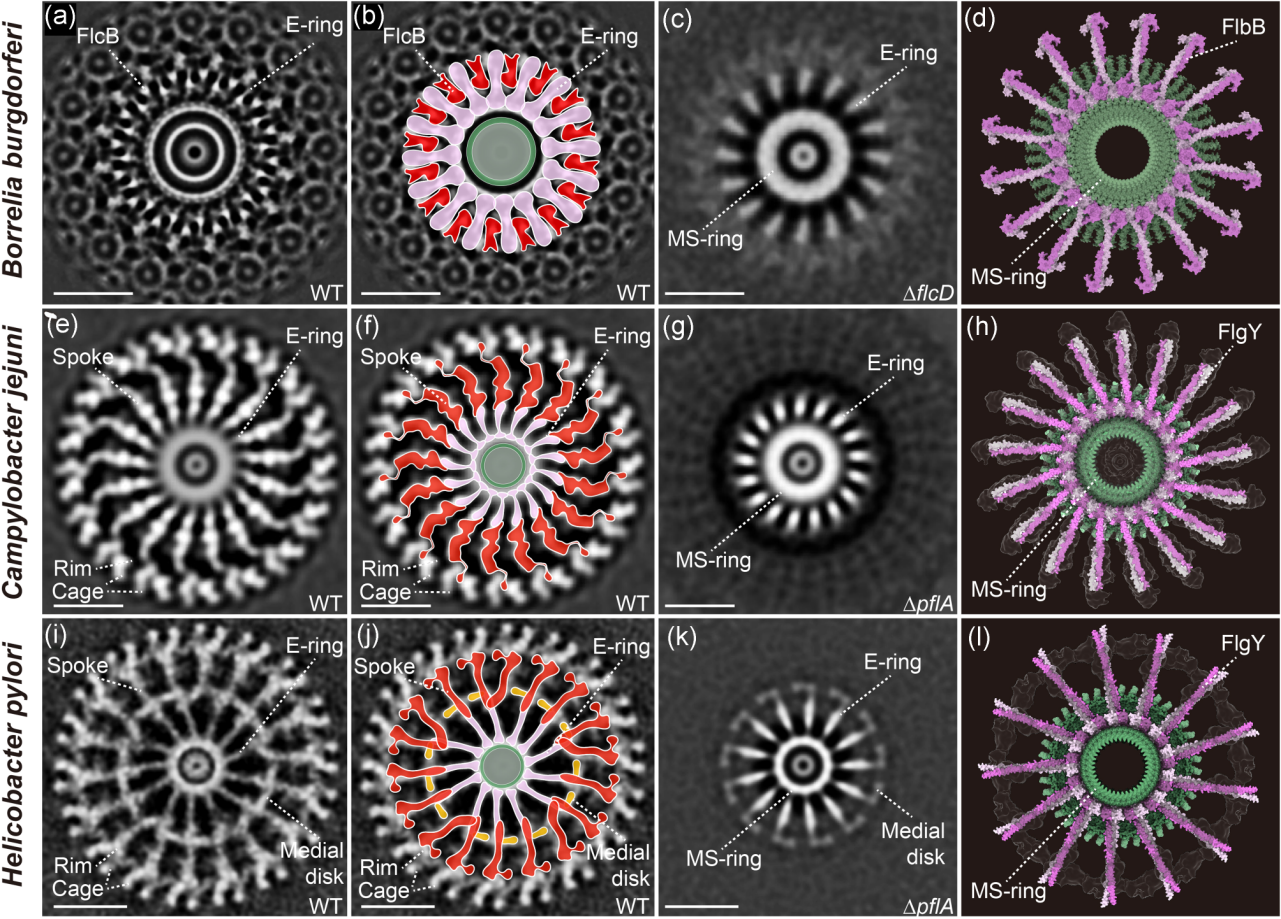
E-ring in flagellar motors of *B. burgdorferi, C. jejuni*, and *H. pylori*. The cryo-ET images are taken from: *B. burgdorferi* (A–D) (Botting et al. 2025), *C. jejuni* (E–H) (Feng et al. 2026), and *H. pylori* (I–L) (Tachiyama et al. 2025). (A–B, E–F, I–J) Cross-sectional view of the wild-type motor and corresponding cartoon. **(C, G, K)** Cross-sectional view of the Δ*flcD*/Δ*pflA* mutant motor, showing the E-ring and MS-ring. **(D, H, L)** Refined structural model of the FlbB/FlgY dimer–based E-ring. Scale bar in A–C, E–G, I–J: 20 nm.

The exact location and structural details of FlbB in the *B. burgdorferi* motor were further examined recently at higher resolution (∼13 Å) using cryo-ET and subtomogram averaging, as well as comparison of wild-type motor to those of mutants lacking the other four collar components (Botting et al. 2025). The new data suggest that FlbB forms a distinctive “hub-and-spoke” structure around the MS-ring, with 16 spokes connected to a circular hub exhibiting 32-fold symmetry (Fig. 3A) (Botting et al. 2025). FlbB is a small protein of 205 amino acids and forms a dimer. The AlphaFold-predicted FlbB structure consists of an N-terminal transmembrane helix, a long α-helical linker region, and a C-terminal globular head domain (Fig. 2). Fitting 16 FlbB dimers predicted by AlphaFold Multimer into the cryo-ET maps of the *B. burgdorferi* motor showed good agreement with the “hub-and-spoke” structure (Botting et al. 2025). Specifically, the C-terminal head domains of FlbB dimers fit well into the hub surrounding the MS-ring; the parallel coiled-coil formed by the long α-helical linker region matched each spoke, and the N-terminal transmembrane helix inserted into the inner membrane at the base of the collar (Botting et al. 2025). Molecular modelling of all five known collar components in the *in situ* motor structure suggest that FlbB acts as a scaffold for the assembly of the other collar components, with FlcB on top of FlbB and FlcC and FlcD positioned adjacent to FlbB (Botting et al. 2025). In addition, electrostatic interactions exist between the FlbB hub and MS-ring, similar to the L/P-rings and central rod, suggesting a possible role for FlbB ring as a bearing that enables stable rotation of the MS-ring (Botting et al. 2025).

## FlgY in *C. jejuni*

We recently sought to identify the component of the E-ring in *C. jejuni* by imaging motor structures of mutants of several novel motility genes identified from our previous TnSeq screens (Gao et al. 2014, Gao et al. 2017). Notably, Tnseq screens using cell invasion and mouse infection identified several genes impacting motility, including genes whose deletion significantly decreased fitness during host interaction but did not change motility in soft agar assays or in liquid medium (Gao et al. 2014, Gao et al. 2017). Among them, only the Δ*flgY* mutant lacked a periplasmic ring structure around the central rod proximal to the inner membrane where the E-ring is observed (Feng et al. 2026). E-ring is not a continuous ring or disk, but rather a small, thin ring around the MS-ring with 17 separate spokes (Fig. 3) (Feng et al. 2026). This “ring-spoke” structure in *C. jejuni* is similar to the “hub-and-spoke” structure formed by FlbB in the *B. burgdorferi* motor, in terms of their overall shape and specific position encircling the MS-ring (Botting et al. 2025, Feng et al. 2026). The main difference is that the radiating spokes in *C. jejuni* are not inserted into the inner membrane but instead interact with 17 additional, longer spokes made of PflA (Fig. 3) (Feng et al. 2026). Consistently, FlgY has a signal peptide at its N-terminus without any transmembrane motif while FlbB possesses a transmembrane motif without signal peptide (Fig. 2).

FlgY shares little sequence homology with FlbB, but they are structurally similar. AlphaFold3 prediction of the FlgY structure shows a long α-helix followed by a globular head domain (Fig. 2). Comparison of the head domains of FlgY and FlbB revealed identical four-membered, right-handed superhelices with a hydrophobic core (Feng et al. 2026). Notably, this head domain shares structural similarity with the N-terminal cytosolic domain of the Mg^2+^ transporter MgtE as well as the armadillo repeat motif (ARM)-like motifs in flagellar rotor protein FliG, which differs from the canonical ARM repeat in terms of helical packing (Hattori et al. 2007, Lynch et al. 2017, Xue et al. 2018, Feng et al. 2026). In addition, like FlbB, FlgY also forms a dimer and the predicted structure of FlgY dimers fit well with the cryo-ET map of the *C. jejuni* motor (Feng et al. 2026). Similar to the FlbB ring, 34 ARM-like domains from 17 FlgY dimers form a ring around the β-collar of the MS-ring, and the dimeric coiled-coil domains point outward to connect with PflA spokes at a 1:1 ratio (Feng et al. 2026).

Interestingly, the *C. jejuni* Δ*flgY* mutant remains motile on soft agar and in liquid medium, although it is defective in cell invasion (Table 1) (Feng et al. 2026). In line with the motility phenotype, the motor structure of the Δ*flgY* mutant only lacks the E-ring, while 17 stator units and the other periplasmic scaffolds remain intact (Feng et al. 2026). Thus, how FlgY and the E-ring contribute to *C. jejuni* motor function remains unclear. Moreover, the E-ring is present in mutants of genes encoding periplasmic scaffold proteins in *C. jejuni* including PflA/PflB/PflC/PflD, FcpM/FcpN/FcpO, as well as FlgP (Beeby et al. 2016, Cohen et al. 2024, Drobnic et al. 2025, Feng et al. 2026). Thus, the assembly of E-ring does not rely on the presence of other periplasmic scaffold proteins examined so far.

Lastly, the E-ring is present in *C. jejuni* Δ*rpoN* mutant as observed by cryo-ET imaging (Feng et al. 2026). RpoN (σ^54^) is an early checkpoint regulator of flagellar assembly in both *C. jejuni* and *H. pylori*, regulating the gene expression of the rod, L-/P-rings, hook and the downstream regulator FliA (σ^28^) (Jagannathan et al. 2001, Lertsethtakarn et al. 2011). Consistent with previous observation of E-ring in Δ*flgA* mutant of *C. crescentus* (Hahnenberger and Shapiro 1988), these results suggest that E-ring assembles very early in flagellar motors, prior to the rod maturation.

## FlgY in *H. pylori*

The FlgY homolog has also been studied in *H. pylori*, an important human pathogen and close relative of *C. jejuni* (Table 1) (Tachiyama et al. 2025). Intriguingly, although *H. pylori* FlgY also forms a similar “ring-spoke” structure around the MS-ring and interact with PflA spokes, there is a symmetry mismatch in the overall structure (Fig. 3) (Tachiyama et al. 2025). FlgY dimers form a 13-fold “ring-spoke” structure and interact with 18-fold PflA spokes, and the PflA spokes extend to the 18 stator units of *H. pylori* (Fig. 3) (Tachiyama et al. 2025). In contrast, in both *B. burgdorferi* and *C. jejuni*, the symmetry of the E-ring matches the number of stator units (16 and 17, respectively), and no symmetry mismatch is observed at the interfaces between the E-ring and other adjacent structures (Botting et al. 2025, Feng et al. 2026). In addition, a 13-fold medial ring of unknown composition is present in the *H. pylori* motor at the interaction interface of FlgY and PflA spokes, and FlgY is required for the formation of the medial ring (Tachiyama et al. 2025).

Unlike the *C. jejuni* Δ*flgY* mutant, *H. pylori* Δ*flgY* is less motile in soft agar than the wild type strain (Table 1) (Tachiyama et al. 2025). Consistent with this phenotype, the stator units and surrounding scaffolds such as the distal ring (corresponding to PflB rim in *C. jejuni*) and cage are less stable in the *H. pylori* Δ*flgY* motor structure, in addition to the absence of E-ring, medial ring and PflA spokes (Tachiyama et al. 2025). Therefore, FlgY forms the E-ring in both *C. jejuni* and *H. pylori*, which radiates from the MS-ring and interacts with longer spokes that further extend to the stator ring. However, FlgY affects motility differently in these two closely related species, likely due to differences in E-ring symmetry relative to the PflA spokes and stator units, and to the presence of the medial ring of unknown composition in *H. pylori*.

## The ubiquity of the E-ring in the *Bacteria* domain

The mysterious component of the E-ring is now revealed to be a single protein: a homolog of FlgY/FlbB. However, due to their large sequence divergence, as seen between FlgY and FlbB, it is difficult to identify homologs of these proteins bioinformatically to assess E-ring conservation across bacterial phyla. Nevertheless, a prominent feature of FlgY and FlbB is their C-terminal ARM-like domain, which is defined as an MgtE_N domain in the Pfam database (PF03448) (Feng et al. 2026). Thus, a practical solution to identify FlgY homologs is to combine sensitive homology searches using the MgtE_N Hidden Markov Model (HMM) profile from Pfam and structure predication by AlphaFold, and considering only those showing structural similarity (Feng et al. 2026). This strategy proved valid in a test run with representative species of α-proteobacteria since it identified MotE from both *S. meliloti* and *C. sphaeroides* as well as CC_2059 from *C. crescentus* (Feng et al. 2026). The apparent structural similarity of MotE to FlgY (Fig. 2), together with previous reports of MotE dimerization, periplasmic localization, and functional relationships with stator complexes (Eggenhofer et al. 2004, Velez-Gonzalez et al. 2024), strongly supports that MotE is a homolog of FlgY. In addition, the MotE-interacting partners MotC and MotK show structural similarity to PflA (which interacts with FlgY) and FlcB (which interacts with FlbB), and they are all enriched in TPR repeats (Eggenhofer et al. 2004, Velez-Gonzalez et al. 2024, Botting et al. 2025, Feng et al. 2026). Thus, MotE likely forms an E-ring in motors of *S. meliloti* and *C. sphaeroides*, and MotC and MotK are homologs despite great sequence and structural divergence. Although CC_2059 in *C. crescentus* has not been studied, it is structurally similar to FlgY and contains an additional transmembrane motif, and its downstream gene product, CC_2058, is structurally similar to PflA (Fig. 2) (Feng et al. 2026). These observations suggest that the E-ring, first identified in *C. crescentus*, is formed by CC_2059, which interacts with CC_2058, similar to the relationship of FlgY-PflA, MotE-MotC/MotK, and FlbB-FlcB.

**Figure 4:**
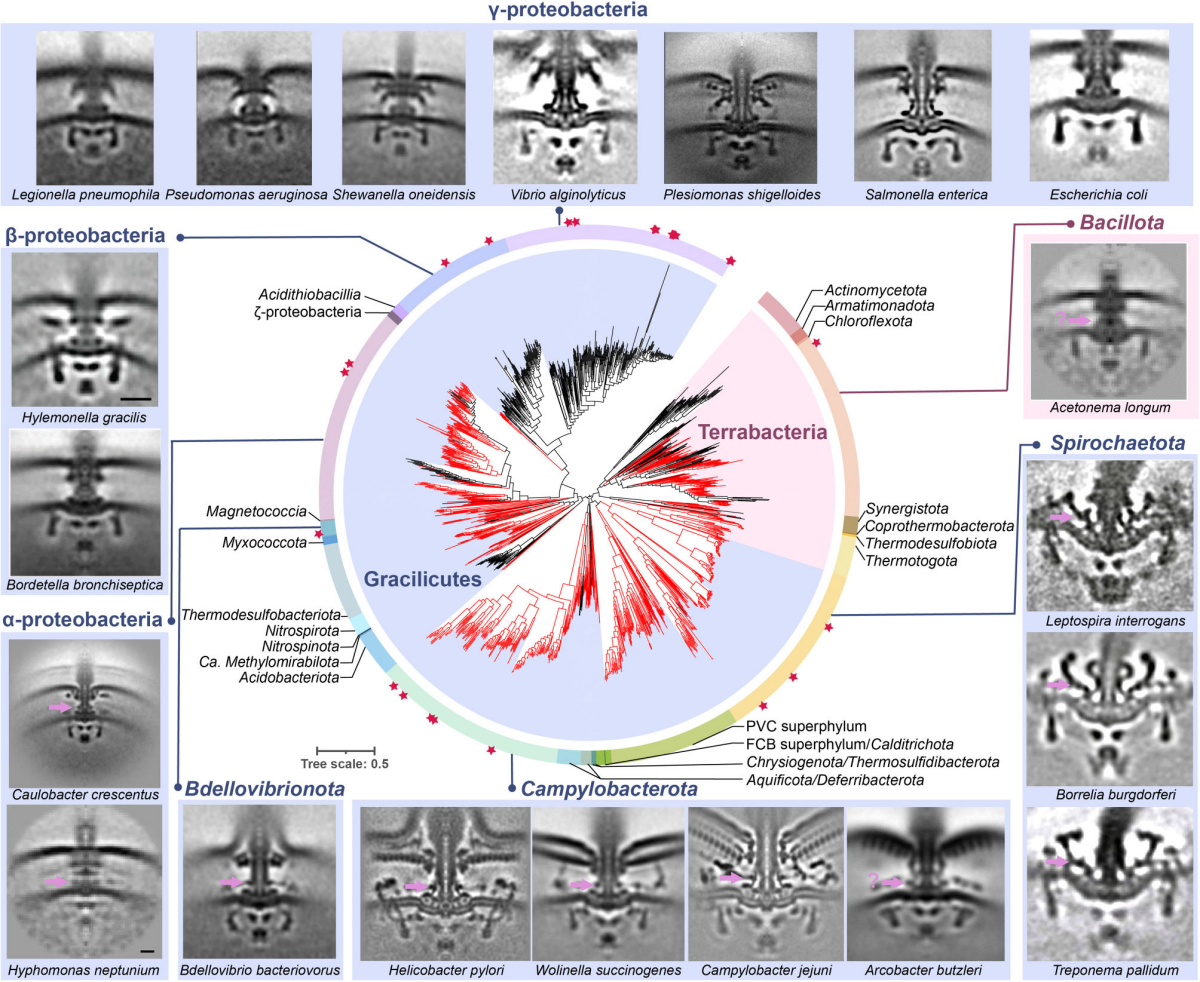
The ubiquity of E-ring in flagellated species of the *Bacteria* domain. The central phylogenetic tree highlighting branches with FlgY homologs is adapted from (Feng et al. 2026). Representative motor structures resolved by cryo-ET are arranged around the periphery, with one representative species from each genus and taxon group labeled at the top of the image. Species marked with stars on the tree directly correspond to the specific cryo-ET images displayed in the outermost ring. Potential E-ring is indicated by arrow and question marks in the images of *Acetonema longum* and *Arcobacter butzleri* mean that the position of E-ring is uncertain, likely due to low resolution. Information on motor structure for each species (clockwise order) were taken from the following references: *Acetonema longum, Hylemonella gracilis, Hyphomonas neptunium* (Chen et al. 2011), *Leptospira interrogans* (Raddi et al. 2012), *B. burgdorferi* (Liu et al. 2009), *Treponema pallidum* (Liu et al. 2010), *Arcobacter butzleri, Wolinella succinogenes, Bdellovibrio bacteriovorus* (Chaban et al. 2018), *C. jejuni* (Feng et al. 2026), *H. pylori* (Tachiyama et al. 2022), *C. crescentus* (Rossmann et al. 2020), *Bordetella bronchiseptica* (Ferreira et al. 2021), *Plesiomonas shigelloides* (Ferreira et al. 2019), *Legionella pneumophila, Pseudomonas aeruginosa, Shewanella oneidensis* (Kaplan et al. 2019), *Vibrio alginolyticus* (Carroll et al. 2020), *S. enterica* (Zhu et al. 2019), *E. coli* (Zhu et al. 2017). *Bacillota* represents the only phylum from Terrabacteria, whereas all the other phyla belong to Gracilicutes.

To assess how many flagellated species have an E-ring in their motors, the above searching strategy was applied across 1 365 representative species with a flagellar gene set, covering a great diversity of bacterial phyla/superphyla (Feng et al. 2026). Surprisingly, 66% of these flagellated species have a FlgY homolog, whereas species lacking FlgY mainly belong to two lineages: the β- and γ-proteobacteria (Fig. 4) (Feng et al. 2026). In particular, FlgY homologs are also found in monoderm species such as *Bacillus subtilis*, whose flagella have been well studied but the apparent FlgY homolog encoded by *ylxF* within the large flagellar gene cluster has been overlooked (Fig. 2). Notably, the identified *flgY* gene is most often located adjacent to *fliJ, fliK*, or both, in many lineages, suggesting conserved synteny and a role in motility (Fig. 2). Moreover, about one-third of FlgY homologs have a signal peptide, whereas the other two-thirds have a transmembrane motif at their N terminus (Feng et al. 2026). It is possible that the transmembrane motifs of some FlgY homologs play important structural roles in the motor, similar to FlbB in *B. burgdorferi* (Botting et al. 2025).

The ubiquity of FlgY homologs and potentially the E-ring in flagellated species has profound evolutionary implications. Recent studies suggest that the root of the bacterial phylogenetic tree lies between Terrabacteria (including monoderm and atypical diderm lineages) and Gracilicutes (including most diderm lineages) (Coleman et al. 2021). In addition, phenotypic reconstruction of the last bacterial common ancestor (LBCA) proposes that it was a flagellated, rod-shaped diderm organism (Coleman et al. 2021). Hence, the wide presence of FlgY homologs in flagellated species of both Terrabacteria and Gracilicutes suggests that the E-ring likely has an ancient origin and evolved in the ancestral motor of the LBCA (Fig. 4) (Feng et al. 2026). Flagellated species without FlgY homologs likely lost this protein during evolution. One may argue that species with FlgY homologs may have obtained this gene by horizontal gene transfer (HGT) or convergent evolution, but either HGT or convergent evolution requires many “gene gain” events to achieve the wide presence of FlgY homologs in modern species while few “gene loss” events fit the parsimony theory in this case. In particular, all species of β- and γ-proteobacteria do not have FlgY homolog and these two bacterial groups form a cluster in the phylogenetic tree, thus it is more likely that *flgY* gene loss happened once in the last common ancestor of β- and γ-proteobacteria (Fig. 4). Furthermore, the classical *E. coli* model with the simplest motor, also a product of horizontal gene transfer from β-proteobacteria rather than inherited from its γ-proteobacteria ancestor (Ferreira et al. 2021), while valuable for providing initial insights into flagella structure-function, cannot serve as a prototype of the ancestral bacterial flagellar motor.

## Concluding remarks

In summary, the E-ring is an ancient and widespread, but previously unappreciated, component of the flagellar nanomachine. We define the E-ring as a “ring and spoke” structure encircling the upper part of the MS-ring in flagellar motors and formed by a single protein, FlgY and its homologs. Although the location and overall shape of E-ring in flagellar motors examined by cryo-ET is conserved, it seems to function differently across diverse species. It is absolutely required for flagellar motility in α-proteobacteria and *B. burgdorferi*, but less so in *H. pylori*, and is not required for *C. jejuni* swimming in soft agar. Thus, the exact role of the E-ring in motor function remains to be elucidated by more systematic and fine-grained motility behavior tests. In addition, these tests should take each species’ native environment into consideration and perhaps not just in domesticated lab strains. Nevertheless, the specific position of the E-ring encircling the MS-ring suggest that it might serve as a bearing to support the rotating MS-ring, similar to the upper L-/P-rings for the rod (Botting et al. 2025, Tachiyama et al. 2025). Differences in motility phenotypes may also be explained by different interactions of the E-ring with other periplasmic scaffolds and stator units in each species. For example, although the E-ring symmetry in most examined species is identical with interacting scaffolds and stator units, there is a symmetry mismatch between the E-ring and its interacting PflA spokes in *H. pylori* (Tachiyama et al. 2025). In addition, whether the E-ring is anchored in the inner membrane may also affect its role in the motor. In the case of FlbB in *B. burgdorferi*, its transmembrane motif may assist its scaffolding role in assembling other collar proteins (Botting et al. 2025). Moreover, FlbB interacts with FlcB in a manner different from the way FlgY interacts with PflA in *C. jejuni*. According to the molecular model, FlcB is on top of the coiled-coil region of E-ring, and other scaffolds such as FlcD are also in close contact with E-ring (Botting et al. 2025), In contrast, in *C. jejuni*, the E-ring overlaps with the PflA spokes at their ends, and no densities are observed atop its coiled-coil region in the cryo-ET map (Feng et al. 2026).

The assembly of the E-ring itself is another interesting question. In *C. jejuni*, the assembly of the E-ring into the motor is independent of any periplasmic scaffolding proteins, such as PflABCD, FlgPQ, or the peripheral cage. Based on RNA-seq and cryo-ET data from *C. jejuni* regulatory mutants, the E-ring assembles very early, before the rod (Feng et al. 2026). The MS-ring is among the earliest assembled parts of the motor (Kaplan et al. 2022, Dornes et al. 2024). It is possible that, once the MS-ring forms, FlgY dimers assemble into a ring around it. Given that the P-ring has a specific chaperone, FlgA, it is reasonable to hypothesize that the E-ring also requires a chaperone to assist its assembly, although this remains to be determined. In addition, how the dimeric ARM-like domains stack to form a ring merits investigation, particularly given that different symmetries have been observed for the E-ring in different species.

Therefore, the story of the E-ring is complex and will continue. Mechanisms of E-ring assembly and function will reveal its versatile role in diverse species. Moreover, the ubiquity of E-ring suggests that the classical *E. coli/S. enterica* model is not a prototype of ancestral motor, which likely possessed an E-ring. Consistently, the diversity in the structure and function of the E-ring across bacterial phyla also points to an ancient origin, i.e. it has had time to diverge.

## References

[bib1] Abram D, Koffler H, Vatter AE. Basal structure and attachment of flagella in cells of Proteus vulgaris. J Bacteriol. 1965;90:1337–54. 10.1128/jb.90.5.1337-1354.1965. 5848332 PMC315823

[bib2] Abramson J, Adler J, Dunger J et al. Accurate structure prediction of biomolecular interactions with AlphaFold 3. Nature. 2024;630:493–500. 10.1038/s41586-024-07487-w. 38718835 PMC11168924

[bib3] Armitage JP, Berry RM. Assembly and dynamics of the bacterial flagellum. Annu Rev Microbiol. 2020;74:181–200. 10.1146/annurev-micro-090816-093411. 32603624

[bib4] Beeby M, Ferreira JL, Tripp P et al. Propulsive nanomachines: the convergent evolution of archaella, flagella and cilia. FEMS Microbiol Rev. 2020;44:253–304. 10.1093/femsre/fuaa006. 32149348

[bib5] Beeby M, Ribardo DA, Brennan CA et al. Diverse high-torque bacterial flagellar motors assemble wider stator rings using a conserved protein scaffold. Proc Nat Acad Sci USA. 2016;113:E1917–1926. 26976588 10.1073/pnas.1518952113 PMC4822576

[bib6] Berg HC, Anderson RA. Bacteria swim by rotating their flagellar filaments. Nature. 1973;245:380–2. 10.1038/245380a0. 4593496

[bib7] Botting JM, Rahman MK, Xu H et al. FlbB forms a distinctive ring essential for periplasmic flagellar assembly and motility in *Borrelia burgdorferi*. PLoS Pathog. 2025;21:e1012812. 10.1371/journal.ppat.1012812. 39777417 PMC11750108

[bib8] Carroll BL, Nishikino T, Guo W et al. The flagellar motor of Vibrio alginolyticus undergoes major structural remodeling during rotational switching. eLife. 2020;9:e61446. 10.7554/eLife.61446. 32893817 PMC7505661

[bib9] Chaban B, Coleman I, Beeby M. Evolution of higher torque in Campylobacter-type bacterial flagellar motors. Sci Rep. 2018;8:97. 10.1038/s41598-017-18115-1. 29311627 PMC5758724

[bib10] Chang Y, Xu H, Motaleb MA et al. Characterization of the flagellar collar reveals structural plasticity essential for spirochete motility. mBio. 2021;12:e0249421. 10.1128/mBio.02494-21. 34809456 PMC8609358

[bib11] Chang Y, Zhang K, Carroll BL et al. Molecular mechanism for rotational switching of the bacterial flagellar motor. Nat Struct Mol Biol. 2020;27:1041–7. 10.1038/s41594-020-0497-2. 32895555 PMC8129871

[bib12] Chen S, Beeby M, Murphy GE et al. Structural diversity of bacterial flagellar motors. EMBO J. 2011;30:2972–81. 10.1038/emboj.2011.186. 21673657 PMC3160247

[bib13] Chevance FF, Hughes KT. Coordinating assembly of a bacterial macromolecular machine. Nat Rev Micro. 2008;6:455–65. 10.1038/nrmicro1887. PMC5963726 18483484

[bib14] Cohen EJ, Drobnic T, Ribardo DA et al. Evolution of a large periplasmic disk in Campylobacterota flagella enables both efficient motility and autoagglutination. Dev Cell. 2024;59:3306–3321.e5. 10.1016/j.devcel.2024.09.008. 39362219 PMC11652260

[bib15] Coleman GA, Davin AA, Mahendrarajah TA et al. A rooted phylogeny resolves early bacterial evolution. Science. 2021;372:eabe0511. 10.1126/science.abe0511. 33958449

[bib16] Deme JC, Johnson S, Vickery O et al. Structures of the stator complex that drives rotation of the bacterial flagellum. Nat Microbiol. 2020;5:1553–64. 10.1038/s41564-020-0788-8. 32929189 PMC7610383

[bib17] DePamphilis ML, Adler J. Fine structure and isolation of the hook-basal body complex of flagella from *Escherichia coli* and Bacillus subtilis. J Bacteriol. 1971;105:384–95. 10.1128/jb.105.1.384-395.1971. 4993325 PMC248366

[bib18] Dornes A, Schmidt LM, Mais CN et al. Polar confinement of a macromolecular machine by an SRP-type GTPase. Nat Commun. 2024;15:5797. 10.1038/s41467-024-50274-4. 38987236 PMC11236974

[bib19] Drobnic T, Cohen EJ, Calcraft T et al. In situ structure of a bacterial flagellar motor at subnanometre resolution reveals adaptations for increased torque. Nat Microbiol. 2025;10:1723–40. 10.1038/s41564-025-02012-9. 40595286 PMC12222027

[bib20] Eggenhofer E, Haslbeck M, Scharf B. MotE serves as a new chaperone specific for the periplasmic motility protein, MotC, in *Sinorhizobium meliloti*. Mol Microbiol. 2004;52:701–12. 10.1111/j.1365-2958.2004.04022.x. 15101977

[bib21] Einenkel R, Qin K, Schmidt J et al. The structure of the complete extracellular bacterial flagellum reveals the mechanism of flagellin incorporation. Nat Microbiol. 2025;10:1741–57. 10.1038/s41564-025-02037-0. 40595287 PMC12221982

[bib22] Fabela S, Domenzain C, De la Mora J et al. A distant homologue of the FlgT protein interacts with MotB and FliL and is essential for flagellar rotation in *Rhodobacter sphaeroides*. J Bacteriol. 2013;195:5285–96. 10.1128/JB.00760-13. 24056105 PMC3837945

[bib23] Feng X, Tachiyama S, He J et al. Structural insights into the assembly and evolution of a complex bacterial flagellar motor. Nat Microbiol. 2026; 11:770–85.,. 10.1038/s41564-025-02248-5. 41513997

[bib24] Ferreira JL, Coleman I, Addison ML et al. The “Jack-of-all-Trades” flagellum from *Salmonella* and *E. coli* was horizontally acquired from an ancestral beta-proteobacterium. Front Microbiol. 2021;12:643180. 10.3389/fmicb.2021.643180. 33859630 PMC8042155

[bib25] Ferreira JL, Gao FZ, Rossmann FM et al. Gamma-proteobacteria eject their polar flagella under nutrient depletion, retaining flagellar motor relic structures. PLoS Biol. 2019;17:e3000165. 10.1371/journal.pbio.3000165. 30889173 PMC6424402

[bib26] Gao B, Lara-Tejero M, Lefebre M et al. Novel components of the flagellar system in epsilonproteobacteria. mBio. 2014;5:e01349–01314. 10.1128/mBio.01349-14. 24961693 PMC4073491

[bib27] Gao B, Vorwerk H, Huber C et al. Metabolic and fitness determinants for in vitro growth and intestinal colonization of the bacterial pathogen *Campylobacter jejuni*. PLoS Biol. 2017;15:e2001390. 10.1371/journal.pbio.2001390. 28542173 PMC5438104

[bib28] Guo W, Zhang S, Park JH et al. Structures of the sheathed flagellum reveal mechanisms of assembly and rotation in Vibrio cholerae. Nat Microbiol. 2025;10:3305–14. 10.1038/s41564-025-02161-x. 41174224 PMC13137154

[bib29] Hahnenberger KM, Shapiro L. Identification of a gene cluster involved in flagellar basal body biogenesis in *Caulobacter crescentus*. J Mol Biol. 1987;194:91–103. 10.1016/0022-2836(87)90718-2. 3039149

[bib30] Hahnenberger KM, Shapiro L. Organization and temporal expression of a flagellar basal body gene in *Caulobacter crescentus*. J Bacteriol. 1988;170:4119–24. 10.1128/jb.170.9.4119-4124.1988. 2842303 PMC211417

[bib31] Hattori M, Tanaka Y, Fukai S et al. Crystal structure of the MgtE Mg2+ transporter. Nature. 2007;448:1072–5. 10.1038/nature06093. 17700703

[bib32] Hernandez-Valle J, Domenzain C, de la Mora J et al. The master regulators of the Fla1 and Fla2 flagella of *Rhodobacter sphaeroides* control the expression of their cognate CheY proteins. J Bacteriol. 2017;199:e00670–16. 10.1128/JB.00670-16. 27956523 PMC5309913

[bib33] Hu H, Santiveri M, Wadhwa N et al. Structural basis of torque generation in the bi-directional bacterial flagellar motor. Trends Biochem Sci. 2022;47:160–72. 10.1016/j.tibs.2021.06.005. 34294545

[bib34] Jagannathan A, Constantinidou C, Penn CW. Roles of rpoN, fliA, and flgR in expression of flagella in Campylobacter jejuni. J Bacteriol. 2001;183:2937–42. 10.1128/JB.183.9.2937-2942.2001. 11292815 PMC99512

[bib35] Johnson RC, Walsh MP, Ely B et al. Flagellar hook and basal complex of *Caulobacter crescentus*. J Bacteriol. 1979;138:984–9. 10.1128/jb.138.3.984-989.1979. 457596 PMC218131

[bib36] Johnson S, Deme JC, Furlong EJ et al. Structural basis of directional switching by the bacterial flagellum. Nat Microbiol. 2024;9: 1282–92. 10.1038/s41564-024-01630-z. 38459206

[bib37] Johnson S, Fong YH, Deme JC et al. Symmetry mismatch in the MS-ring of the bacterial flagellar rotor explains the structural coordination of secretion and rotation. Nat Microbiol. 2020;5:966–75. 10.1038/s41564-020-0703-3. 32284565 PMC7320910

[bib38] Johnson S, Furlong EJ, Deme JC et al. Molecular structure of the intact bacterial flagellar basal body. Nat Microbiol. 2021;6:712–21. 10.1038/s41564-021-00895-y. 33931760 PMC7610862

[bib39] Kaplan M, Ghosal D, Subramanian P et al. The presence and absence of periplasmic rings in bacterial flagellar motors correlates with stator type. eLife. 2019;8:e43487. 10.7554/eLife.43487. 30648971 PMC6375700

[bib40] Kaplan M, Oikonomou CM, Wood CR et al. Novel transient cytoplasmic rings stabilize assembling bacterial flagellar motors. EMBO J. 2022;41:e109523. 10.15252/embj.2021109523. 35301732 PMC9108667

[bib41] Kawamoto A, Miyata T, Makino F et al. Native flagellar MS ring is formed by 34 subunits with 23-fold and 11-fold subsymmetries. Nat Commun. 2021;12:4223. 10.1038/s41467-021-24507-9. 34244518 PMC8270960

[bib42] Krogh A, Larsson B, von Heijne G et al. Predicting transmembrane protein topology with a hidden Markov model: application to complete genomes. J Mol Biol. 2001;305:567–80. 10.1006/jmbi.2000.4315. 11152613

[bib43] Lertsethtakarn P, Ottemann KM, Hendrixson DR. Motility and chemotaxis in *Campylobacter* and *Helicobacter*. Annu Rev Microbiol. 2011;65:389–410. 10.1146/annurev-micro-090110-102908. 21939377 PMC6238628

[bib44] Liu J, Howell JK, Bradley SD et al. Cellular architecture of Treponema pallidum: novel flagellum, periplasmic cone, and cell envelope as revealed by cryo electron tomography. J Mol Biol. 2010;403:546–61. 10.1016/j.jmb.2010.09.020. 20850455 PMC2957517

[bib45] Liu J, Lin T, Botkin DJ et al. Intact flagellar motor of *Borrelia burgdorferi* revealed by cryo-electron tomography: evidence for stator ring curvature and rotor/C-ring assembly flexion. J Bacteriol. 2009;191:5026–36. 10.1128/JB.00340-09. 19429612 PMC2725586

[bib46] Liu X, Tachiyama S, Zhou X et al. Bacterial flagella hijack type IV pili proteins to control motility. Proc Natl Acad Sci USA. 2024;121:e2317452121. 10.1073/pnas.2317452121. 38236729 PMC10823254

[bib47] Lynch MJ, Levenson R, Kim EA et al. Co-Folding of a FliF-FliG Split Domain Forms the Basis of the MS:c Ring Interface within the Bacterial Flagellar Motor. Structure. 2017;25:317–28. 10.1016/j.str.2016.12.006. 28089452 PMC5387689

[bib48] Merino S, Tomas JM. The FlgT protein is involved in Aeromonas hydrophila polar flagella stability and not affects anchorage of lateral flagella. Front Microbiol. 2016;7:1150. 10.3389/fmicb.2016.01150. 27507965 PMC4960245

[bib49] Molero R, Wilhelms M, Infanzon B et al. Aeromonas hydrophila motY is essential for polar flagellum function, and requires coordinate expression of motX and Pom proteins. Microbiology (Reading). 2011;157:2772–84. 10.1099/mic.0.049544-0. 21737499

[bib50] Moon KH, Zhao X, Manne A et al. Spirochetes flagellar collar protein FlbB has astounding effects in orientation of periplasmic flagella, bacterial shape, motility, and assembly of motors in *Borrelia burgdorferi*. Mol Microbiol. 2016;102:336–48. 10.1111/mmi.13463. 27416872 PMC5055450

[bib51] Moon KH, Zhao X, Xu H et al. A tetratricopeptide repeat domain protein has profound effects on assembly of periplasmic flagella, morphology and motility of the lyme disease spirochete *Borrelia burgdorferi*. Mol Microbiol. 2018;110:634–47. 10.1111/mmi.14121. 30303576 PMC6218285

[bib52] Motaleb MA, Liu J, Wooten RM. Spirochetal motility and chemotaxis in the natural enzootic cycle and development of Lyme disease. Curr Opin Microbiol. 2015;28:106–13. 10.1016/j.mib.2015.09.006. 26519910 PMC4688064

[bib53] Murphy GE, Leadbetter JR, Jensen GJ. In situ structure of the complete Treponema primitia flagellar motor. Nature. 2006;442:1062–4. 10.1038/nature05015. 16885937

[bib54] Perez-Gonzalez C, Domenzain C, Poggio S et al. Characterization of FlgP, an essential protein for flagellar assembly in *Rhodobacter sphaeroides*. J Bacteriol. 2019;201:e00752–18. 10.1128/JB.00752-18. 30559113 PMC6379573

[bib55] Raddi G, Morado DR, Yan J et al. Three-dimensional structures of pathogenic and saprophytic Leptospira species revealed by cryo-electron tomography. J Bacteriol. 2012;194:1299–306. 10.1128/JB.06474-11. 22228733 PMC3294836

[bib56] Rossmann FM, Beeby M. Insights into the evolution of bacterial flagellar motors from high-throughput in situ electron cryotomography and subtomogram averaging. Acta Crystallogr D Struct Biol. 2018;74:585–94. 10.1107/S2059798318007945. 29872008 PMC6096493

[bib57] Rossmann FM, Hug I, Sangermani M et al. In situ structure of the *Caulobacter crescentus* flagellar motor and visualization of binding of a CheY-homolog. Mol Microbiol. 2020;114:443–53. 10.1111/mmi.14525. 32449846 PMC7534056

[bib58] San Martin F, Fule L, Iraola G et al. Diving into the complexity of the spirochetal endoflagellum. Trends Microbiol. 2023;31:294–307. 10.1016/j.tim.2022.09.010. 36244923

[bib59] Santiveri M, Roa-Eguiara A, Kuhne C et al. Structure and function of stator units of the bacterial flagellar motor. Cell. 2020;183:244–257.e16. 10.1016/j.cell.2020.08.016. 32931735

[bib60] Singh PK, Sharma P, Afanzar O et al. CryoEM structures reveal how the bacterial flagellum rotates and switches direction. Nat Microbiol. 2024;9:1271–81. 10.1038/s41564-024-01674-1. 38632342 PMC11087270

[bib61] Stallmeyer MJ, Hahnenberger KM, Sosinsky GE et al. Image reconstruction of the flagellar basal body of *Caulobacter crescentus*. J Mol Biol. 1989;205:511–8. 10.1016/0022-2836(89)90222-2. 2926815

[bib62] Sultan SZ, Sekar P, Zhao X et al. Motor rotation is essential for the formation of the periplasmic flagellar ribbon, cellular morphology, and *Borrelia burgdorferi* persistence within Ixodes scapularis tick and murine hosts. Infect Immun. 2015;83:1765–77. 10.1128/IAI.03097-14. 25690096 PMC4399055

[bib63] Szymanski CM, Gaynor EC. How a sugary bug gets through the day: recent developments in understanding fundamental processes impacting Campylobacter jejuni pathogenesis. Gut Microbes. 2012;3:135–44. 10.4161/gmic.19488. 22555465 PMC3370946

[bib64] Tachiyama S, Chan KL, Liu X et al. The flagellar motor protein FliL forms a scaffold of circumferentially positioned rings required for stator activation. Proc Natl Acad Sci USA. 2022;119:e2118401119. 10.1073/pnas.2118401119. 35046042 PMC8794807

[bib65] Tachiyama S, Rosinke K, Khan MF et al. FlgY, PflA, and PflB form a spoke-ring network in the high-torque flagellar motor of *Helicobacter pylori*. Proc Natl Acad Sci USA. 2025;122:e2421632122. 10.1073/pnas.2421632122. 40261933 PMC12054838

[bib66] Tan J, Zhang L, Zhou X et al. Structural basis of the bacterial flagellar motor rotational switching. Cell Res. 2024;34:788–801. 10.1038/s41422-024-01017-z. 39179739 PMC11528121

[bib67] Tan J, Zhang X, Wang X et al. Structural basis of assembly and torque transmission of the bacterial flagellar motor. Cell. 2021;184:2665–2679.e19. 10.1016/j.cell.2021.03.057. 33882274

[bib68] Teufel F, Almagro Armenteros JJ, Johansen AR et al. SignalP 6.0 predicts all five types of signal peptides using protein language models. Nat Biotechnol. 2022;40:1023–5. 10.1038/s41587-021-01156-3. 34980915 PMC9287161

[bib69] Velez-Gonzalez F, Marcos-Vilchis A, Vega-Baray B et al. Rotation of the Fla2 flagella of *Cereibacter sphaeroides* requires the periplasmic proteins MotK and MotE that interact with the flagellar stator protein MotB2. PLoS One. 2024;19:e0298028. 10.1371/journal.pone.0298028. 38507361 PMC10954123

[bib70] Wadhwa N, Berg HC. Bacterial motility: machinery and mechanisms. Nat Rev Micro. 2022;20:161–73. 10.1038/s41579-021-00626-4. 34548639

[bib71] Wilson ML, Macnab RM. Co-overproduction and localization of the *Escherichia coli* motility proteins motA and motB. J Bacteriol. 1990;172:3932–9. 10.1128/jb.172.7.3932-3939.1990. 2193926 PMC213376

[bib72] Wunder EA Jr, Figueira CP, Benaroudj N et al. A novel flagellar sheath protein, FcpA, determines filament coiling, translational motility and virulence for the Leptospira spirochete. Mol Microbiol. 2016;101:457–70. 10.1111/mmi.13403. 27113476 PMC4979076

[bib73] Xu H, He J, Liu J et al. BB0326 is responsible for the formation of periplasmic flagellar collar and assembly of the stator complex in *Borrelia burgdorferi*. Mol Microbiol. 2020;113:418–29. 10.1111/mmi.14428. 31743518 PMC7178549

[bib74] Xu H, Hu B, Flesher DA et al. BB0259 encompasses a peptidoglycan lytic enzyme function for proper assembly of periplasmic flagella in *Borrelia burgdorferi*. Front Microbiol. 2021;12:692707. 10.3389/fmicb.2021.692707. 34659138 PMC8517470

[bib75] Xue C, Lam KH, Zhang H et al. Crystal structure of the FliF-FliG complex from *Helicobacter pylori* yields insight into the assembly of the motor MS-C ring in the bacterial flagellum. J Biol Chem. 2018;293:2066–78. 10.1074/jbc.M117.797936. 29229777 PMC5808767

[bib76] Yamaguchi T, Makino F, Miyata T et al. Structure of the molecular bushing of the bacterial flagellar motor. Nat Commun. 2021;12:4469. 10.1038/s41467-021-24715-3. 34294704 PMC8298488

[bib77] Zamba-Campero M, Soliman D, Yu H et al. Broadly conserved FlgV controls flagellar assembly and *Borrelia burgdorferi* dissemination in mice. Nat Commun. 2024;15:10417. 10.1038/s41467-024-54806-w. 39614093 PMC11607428

[bib78] Zhao X, Zhang K, Boquoi T et al. Cryoelectron tomography reveals the sequential assembly of bacterial flagella in *Borrelia burgdorferi*. Proc Natl Acad Sci USA. 2013;110:14390–5. 10.1073/pnas.1308306110. 23940315 PMC3761569

[bib79] Zhu S, Nishikino T, Hu B et al. Molecular architecture of the sheathed polar flagellum in Vibrio alginolyticus. Proc Natl Acad Sci USA. 2017;114:10966–71. 10.1073/pnas.1712489114. 28973904 PMC5642721

[bib80] Zhu S, Nishikino T, Kojima S et al. The vibrio H-Ring facilitates the outer membrane penetration of the polar sheathed flagellum. J Bacteriol. 2018;200:e00387–18. 10.1128/JB.00387-18. 30104237 PMC6182240

[bib81] Zhu S, Schniederberend M, Zhitnitsky D et al. In situ structures of polar and lateral flagella revealed by cryo-electron tomography. J Bacteriol. 2019;201:e00117–00119. 10.1128/JB.00117-19. 31010901 PMC6560136

